# Effects of Attention to Auditory Motion on Cortical Activations during Smooth Pursuit Eye Tracking

**DOI:** 10.1371/journal.pone.0007110

**Published:** 2009-09-22

**Authors:** Oliver Baumann, Mark W. Greenlee

**Affiliations:** 1 Queensland Brain Institute, The University of Queensland, Brisbane, Queensland, Australia; 2 Department of Experimental Psychology, University of Regensburg, Regensburg, Germany; Victoria University of Wellington, New Zealand

## Abstract

**Background:**

In contrast to traditional views that consider smooth pursuit as a relatively automatic process, evidence has been reported for the importance of attention for accurate pursuit performance. However, the exact role that attention might play in the maintenance of pursuit remains unclear.

**Methodology/Principal Findings:**

We analysed the neuronal activity associated with healthy subjects executing smooth pursuit eye movements (SPEM) during concurrent attentive tracking of a moving sound source, which was either in-phase or in antiphase to the executed eye movements. Assuming that attentional resources must be allocated to the moving sound source, the simultaneous execution of SPEM and auditory tracking in diverging directions should result in increased load on common attentional resources. By using an auditory stimulus as a distractor rather then a visual stimulus we guaranteed that cortical activity cannot be caused by conflicts between two simultaneous visual motion stimuli. Our results revealed that the smooth pursuit task with divided attention led to significantly higher activations bilaterally in the posterior parietal cortex and lateral and medial frontal cortex, presumably containing the parietal, frontal and supplementary eye fields respectively.

**Conclusions:**

The additional cortical activation in these areas is apparently due to the process of dividing attention between the execution of SPEM and the covert tracking of the auditory target. On the other hand, even though attention had to be divided the attentional resources did not seem to be exhausted, since the identification of the direction of the auditory target and the quality of SPEM were unaffected by the congruence between visual and auditory motion stimuli. Finally, we found that this form of task-related attention modulated not only the cortical pursuit network in general but also affected modality specific and supramodal attention regions.

## Introduction

Human and non-human primates use smooth pursuit eye movements to ensure that the image of an object of interest falls and remains on or near the fovea. Although it has been argued [1] that smooth pursuit eye movements (SPEM) are executed automatically, and thus do not depend on attention, other authors have found evidence for the importance of attention for accurate pursuit performance [2]–[4]. So far most of this research has concentrated on the close relationship between saccadic eye movements and visual attention [5]–[9], but comparatively few studies have explored the role of attention in SPEM. Many of the studies that have, tend to focus on the role of attention in pursuit initiation [10]–[12]. The exact role that attention might play in the maintenance of pursuit remains unclear, but one possibility is that it facilitates motion processing. The suggestion that attentionally mediated deficits in motion processing may lead to pursuit impairments is corroborated by research in patients with schizophrenia. Schizophrenia is associated with severe impairments in smooth pursuit [13] and several studies suggest that these impairments may be linked to impairments in visual motion processing [14]–[15].

The few studies that have explored the role of attention during pursuit maintenance have produced conflicting results. Some recent research appears to support the notion that smooth pursuit is a relatively automatic behaviour [1], [16]. These studies found that performing a secondary task led to improved smooth pursuit [16], whereas other [17], [18] studies have reported that secondary tasks can lead to varying degrees of impairment (i.e. increases in blinks, saccadic eye movements and “non-tracking episodes” during pursuit).

One critical property of attention is that it can be directed to specific positions in space [19]. If attentional resources play a role in the control of SPEM, those concerned with the localization of information within external space might be expected to be particularly relevant. Hutton and Tegaly [3] tested this assumption by having subjects judge either the pitch or the location of a sound, but could not find a difference between the spatial and non-spatial task, despite the confirmation of a general impact of the secondary task on the quality of the pursuit eye movements. One possible reason for their failure to observe differential effects of the two tasks is that the spatial distractor task did not place sufficient demands on spatial attention. The tones were also presented through headphones, which leads to a perception of the tones being located in the subject's head. Using externally created sounds and a condition that requires more than very brief shifts of attention, may result in greater demands on spatial attention. Hence, the research results described above do not unequivocally establish a role of attention in the control of SPEM. Moreover, the nature of the secondary task may be an important determinant of the type of effect observed.

Regarding the neuronal correlates of attentional process on eye movements, most of the previous studies investigated attentional processes during saccades to a peripheral target, comparing effects of attention shifts without saccades (so-called covert shifts of attention, with gaze fixation) to attention shifts in parallel with saccades (overt shifts of attention). A common result of these studies is that the cortical network for directing the focus of visual attention seems to overlap widely with the network for saccadic eye movements. These findings led to the conclusion that covert attention shifts and saccades are subserved by similar neural mechanisms.

The human cortical pursuit network has been studied quite extensively using fMRI and it is known that the performance of pursuit eye movements induces activations in a set of cortical regions known to also subserve the control of saccadic eye movements, namely the frontal eye fields, the supplementary eye fields, the parietal eye fields, precuneus, and middle temporal cortex (MT/MST) [20]–[26]. Regarding the interaction between the smooth pursuit network and attention, Culham and colleagues [27] showed that, during fixation of a stationary target, attentive tracking and attentive saccadic shifts activated the same cortical regions, namely the intraparietal sulcus, postcentral sulcus, superior parietal lobule, cuneus, frontal eye fields and precentral sulcus, but they did not investigate attention directly during SPEM. To our knowledge there is only one fMRI study [4] that investigated continuous, covert, attentive tracking in relation to SPEM. In this study it was explored whether gaze and attention can be divided during pursuit. Ohlendorf and colleagues presented a sinusoidally moving visual target for pursuit and simultaneously a stationary visual target for fixation. Gaze could be directed to the pursuit target and attention to the fixation target or vice versa, or gaze and attention were directed to the same (moving or stationary) target. It was found that gaze (overt) and attentive (covert) pursuit activated the same cortical oculomotor network. Gaze (overt) pursuit showed higher activations than attentive (covert) pursuit. Activations, specific to the dissociation of attention from gaze and independent of eye movements, were found solely in the posterior parietal cortex. The authors concluded that attention control during gaze pursuit and gaze fixation occurs within the cortical SPEM network, supporting the premotor theory of attention [28].

We wanted to further explore the relationship between SPEM and attention by recording the neuronal activity associated with SPEM during concurrent attentive tracking of a moving sound source, which can be either in-phase or in antiphase to the executed eye movements. The rationale behind this approach is that our task requires some attentional resources to be allocated to the moving sound source. If visually guided smooth pursuit and the auditory tracking task share a common attentional resource, having to perform SPEM to a visual target and auditory tracking of a target moving in diverging directions should result in increased load on the attentional resources. The tracking of a moving secondary stimulus might also place higher demands on spatial attention than performing a discrimination task on stationary targets. There is already an extensive literature detailing cross modal interactions in spatial attention [29], [30], particularly between vision and audition and these interactions might extend to the control of pursuit eye movements.

Visual motion processing for perception and smooth pursuit are intertwined [31] whereas auditory signals are apparently unable to generate motion signals capable of supporting SPEM [32]. Using an auditory stimulus as a distractor rather than a visual stimulus has the advantage that potential impairments in SPEM and associated changes in cortical activity cannot solely be caused by conflicts between two simultaneous visual motion signals. On the other hand, sustained and accurate SPEM clearly requires visual signals that specify the relative retinal motion of a visual target [33]. A moving auditory target is therefore less likely to interfere with the basic mechanisms driving SPEM, and should therefore be better suited for testing isolated effects of divided spatial attention on brain areas controlling SPEM. In particular, we wanted to answer the question, to what extent the covert tracking of an auditory target during SPEM influences the activation of the cortical pursuit network and if divided attention modulates the cortical pursuit network in general or if attention activates specific regions in addition to the SPEM system. Augmentation in activity would indicate a compensatory recruitment. On the other hand, decreased brain activity in eye movement control centers could be interpreted as a direct consequence of the division of attentional resources. On the cortical level, we expect to find differences in the activation of regions-of-interest in the frontal, supplementary, and parietal eye fields, the cingulate gyrus, the areas MT and MST, and the precuneus.

## Results

### Eye movements and behavioural data

**Figure 1:**
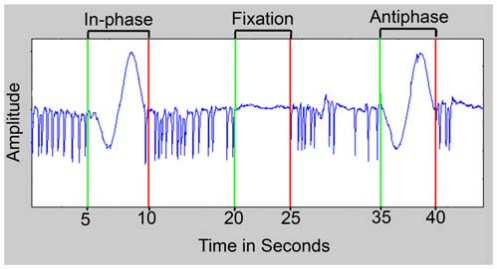
Eye movement data. Original eye movement traces for all three conditions: “SPEM with in-phase auditory target”, “Fixation with moving auditory target” and “SPEM with antiphase auditory target”.

The maximum deviations from fixation were <1° in all conditions, which were due to baseline drifts and noise. As in the training session outside the scanner, all subjects were able to maintain stable fixation. The SPEM were further qualitatively inspected; it was evident that the subjects were able to follow the visual targets with their eyes smoothly in both conditions, never made initial eye movements in the wrong direction, and they did not execute explorative saccades. To monitor and analyze possibly small differences in gain and occurrences of very small catch-up saccades (∼1°), the quality (due to baseline drifts and noise) and extent (only 1 cycle per trial) of the eye movements recorded during the fMRI session was not sufficient. Original eye movement traces for all three experimental conditions are shown in Figure 1.

With respect to the direction judgments of the moving sound source, a majority of the subjects had a hit-rate of 100% and all of them had a hit-rate of at least 95%. The mean hit-rate was 99.7% for the condition “Fixation with moving auditory target”, 100% for the condition “SPEM with in-phase auditory target”, and 99.5% for the condition “SPEM with antiphase auditory target”. Because of this ceiling effect we refrained from conducting statistical comparisons. The mean response time was 796 ms (SE = 165 ms) for the condition “Fixation with moving auditory target”, 778 ms (SE = 156 ms) for the condition “SPEM with in-phase auditory target”, and 814 ms (SE = 162 ms) for the condition “SPEM with antiphase auditory target”. Response times were slightly longer in the condition with an incongruent auditory source, but the difference between the two auditory conditions was not statistically significant (Wilcoxon, p>0.05). Because subjects were instructed to wait until the end of stimulation to respond, these results were expected.

### FMRI data

**Figure 2:**
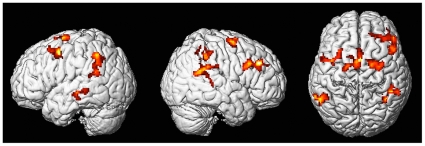
FMRI data. Three-dimensional rendered MR images showing mean BOLD activity from the whole brain analysis in the contrast “SPEM with antiphase auditory target > SPEM with in-phase auditory target”. T-values are overlaid onto a rendered MNI-normalized template. Activated voxels buried within sulci are projected onto the cortical surface.

Pursuit eye movements towards the visual target activated, irrespective of the direction of the auditory sound source, the well-established pursuit network, which included cuneus, precuneus, MT, posterior parietal cortex, supplementary eye fields, and frontal eye fields. However, the condition “SPEM with sound source in antiphase” led to a clear general tendency for more cortical activation in the whole SPEM system and significantly higher activations in the superior temporal cortex, which anatomically corresponds to the motion sensitive auditory cortex [34], [35]. The direct statistical comparison “SPEM with sound source in antiphase > SPEM with sound source in-phase” revealed increased activity in (1) posterior parietal (bilateral inferior parietal lobule and supramarginal gyrus, predominantly in Brodmann area 40, presumably containing the parietal eye fields), (2) bilateral in lateral and medial frontal cortex (Brodmann area 6, presumably containing the frontal and supplementary eye fields), as well as (3) bilaterally in the superior temporal gyri, presumably containing the secondary auditory cortex. We further observed increased activation in the right middle frontal gyrus (corresponding to Brodmann area 9; see Table 1 and Figure 2). We did not observe any significant activations for the reversed contrast (in-phase > antiphase).

**Table 1:** MNI coordinates of the maximum t-value within each cluster for the contrast “SPEM with antiphase auditory target > SPEM with in-phase auditory target”.

Region	Hemisphere	Brodmann area	MNI Coordinates (X, Y, Z)	T Value (cluster size in number of voxels)
MFG/SFG/MedFG	R	9	46, 20, 34	5.10 (553)
STG/SMG/IPL	L	40/22	−48, −40, 16	4.89 (476)
MFG/SFG/MedFG	R	6	18, −4, 64	4.86 (284)
CinG/SFG/MedFG	L/R	6/32	−18, −6, 72	4.83 (536)
STG/IPL/SMG	R	40/22	54, −38, 28	4.53 (407)
IPL/SPL/PostcG	R	40	44, −42, 48	4.53 (360)
MFG/SFG	L	6/8	−30, 8, 52	4.49 (268)

Spatial coordinates of the local maxima in the group analysis, showing significant activations (p≤0.05, corrected for multiple comparisons, height threshold t = 3) for the contrast “SPEM with antiphase auditory target > SPEM with in-phase auditory target”. Abbreviations: CinG =  cingulate gyrus, L =  left hemisphere, MedFG =  medial frontal gyrus, MFG =  middle frontal gyrus, PostcG =  postcentral gyrus, R =  right hemisphere, SFG =  superior frontal gyrus, SMG =  supramarginal gyrus, SPL =  superior parietal lobule, STG =  superior temporal gyrus.

## Discussion

Irrespective of the tasks to divide or to focus attention and gaze directions, our fMRI data revealed that SPEM always activated a similar cortical network. However, the smooth pursuit task with divided attention led to a general tendency for more cortical activation in the whole SPEM system and significantly higher activations bilateral in the posterior parietal cortex, and lateral and medial frontal cortex, presumably containing the parietal, frontal and supplementary eye fields, respectively. The additional cortical activation in these areas is assumed to be due to the process of dividing attention between the execution of SPEM and the covert tracking of the auditory target. Thus, additional processing appears to be required to track an auditory target while maintaining smooth pursuit on a visual target. Our data indicate that subjects successfully divided their attention since they were able to execute SPEM in a normal way, and subjects did not exhibit a decreased ability in identifying the direction of the sound source. It could be argued that subjects only needed to attend to the moving sound source for several hundred milliseconds at the beginning of each trial, since this initial information would have been sufficient to perform the task. Nevertheless, the fact that the smooth pursuit task with incongruently moving sound source evoked more neural activity indicates that this condition placed higher demands on the subjects' spatial attention network. However, we asked each subjects at the end of the scan session if they used attention to track the sound source throughout the trial or if they only attended to the moving sound source at the beginning of each trial. Our queries revealed that all subjects stated that they continued to attend to the moving sound source even after they identified the initial direction of its movement, independent of the experimental condition.

A previous study [4], in which subjects' gaze was directed to a visual pursuit target and attention to a visual fixation target or vice versa, reported increased activation to be restricted to the superior posterior parietal cortex in conditions where a dissociation between the focus of attention and gaze eye movements took place. We also found increased parietal activity under a condition of divided attention and independent of eye movements, but the activation clusters were situated more inferior to that reported by Ohlendorf et al [4], corresponding to the location of the parietal eye fields in humans [36]. Further we detected significantly increased activity also in the frontal and supplementary eye fields. On the other hand, the posterior parietal cortex, the lateral superior frontal cortex and the medial frontal cortex are all known, apart for their function for controlling SPEM, to play a role in attentional control. The posterior parietal cortex and especially the intraparietal sulcus have long been known for their role in spatial and non-spatial attention [37], [38] and increased activity in this region has been found before, under conditions of divided attention [39]. Similarly, the frontal eye fields, which, apart for their role in eye movement control, are known to be involved in spatial shifts of attention and covert motion tracking [27], [40]. Finally, the supplementary eye fields are thought to play an executive function for eye movement control and are believed to be involved in oculomotor control during response conflict [41]. These regions might also code for orientation in space and the preparation of motor action [42]. Taken together, these results underline that SPEM and attention are very tightly yoked processes that rely on substantially overlapping neural substrates [5], [43], [44]. Nevertheless, the increased activity in these brain regions caused by the incongruent condition of our task suggests that SPEM depends on attention and is thus not executed automatically. For the execution of visual pursuit the observer needs to first attend to the moving object, to then process its velocity and then prepare a motor signal to allow smooth tracking of the target. A simultaneous spatial tracking task puts extra demands on the SPEM system evident through elevated levels of activity in these brain regions. By using an auditory secondary moving target instead of a visual one it is unlikely that these elevated levels of activity are solely due to conflicting motion signals, since auditory signals alone are unable to generate motion signals capable of supporting SPEM [32].

Interestingly, during the incongruent condition we also detected a more pronounced activation in areas suspected to be involved in the processing of auditory motion [34], [35]. It is plausible to assume that, when the sound source and the visual target move in different directions, more attention is directed not only on the execution of SPEM, but also on the evaluation of the auditory signal. This is a noteworthy observation, since on first hand one would expect a diminished activation pattern in auditory cortex due to the division of attentional resources. Finally, we observed increased activation in the right middle frontal gyrus (corresponding to Brodmann area 9), which has been linked do divided attention [45], sustained attention [46] and response inhibition [47].

In conclusion, our study shows that attention is necessary for the performance of SPEM. This conclusion is supported by the observation that elevated cortical activity occurs during SPEM when subjects divide their attention between moving visual and auditory targets. On the other hand, the attentional resources did not seem to be exhausted, since a drop in behavioural performance would then be expected. The behavioural data shows that under the incongruent condition there was no drop in performance, neither in identification of the direction of the auditory target nor in the quality of SPEM. As a final point, we found that attention modulates not only the cortical pursuit network in general but also affects modality specific (i.e. the auditory cortex) and other supramodal attention regions.

## Methods

### Subjects

Subjects were 19 right-handed volunteers (13 female) who gave their informed written consent to procedures approved by the Regensburg University's ethics committee. The subjects' ages ranged between 18 and 35 years (mean 22 years). None of the subjects had a history of neurological or psychiatric disorders. All subjects were free of hearing and visual impairments. All subjects participated in a training session to practice the task. Subjects' performance was assessed in the psychophysical laboratory prior to imaging to exclude individuals who could not fulfill the criteria described above.

### Visual Stimulation

The subjects were positioned supine in the scanner with their head tightly secured in the headcoil to minimize head movement. They viewed the stimuli with a mirror that reflected the image from a projection screen placed at the head of the subject in the end of the scanner gantry. The image subtended 26° of visual angle horizontally and 20° of visual angle vertically (1024×768 pixels) at a viewing distance of 70 cm. The stimuli were digital movies created with Matlab (Version 6.5). The visual target was a small white square (0.4° visual angle, 80 cd/m^2^) on black background (1.25 cd/m^2^). In the condition with SPEM the target moved with a sinusoidal velocity profile along the horizontal axis starting from the centre. The left and right turning points were 24° visual angle from each other. The maximum speed of 15.1°/s was reached by the dot in the screen centre. A full cycle lasted 5 s. In the stationary condition the fixation dot was presented centrally for 5 s.

### Auditory Stimulation

The moving sound was Gaussian white noise which was convolved with a generic head-related transfer function for positions±12° of azimuth angle, in discrete steps of 1°. The sounds were smoothed by a hanning window to create the impression of a smoothly moving sound source. The virtual sound source had the same sinusoidal velocity profile as the moving fixation dot. The acoustic noise was presented using a Soundblaster soundcard, MR Confon amplifier, and MRI-compatible sound-dampening headphones (MR Confon, GmbH, Magdeburg, Germany). The sound pressure level (SPL) of the auditory noise stimuli was 76 dB(A).

### Audiovisual Stimulation

The visual and auditory stimuli were merged together using an audiovisual editing program (FX RESound, Hepple, Inc., Hewitt, Texas), leading to 5-s episodes of audiovisual digital movies. Overall, three combinations of stimuli were constructed containing either a moving or stationary fixation dot. The fixation dot moved in-phase or in antiphase with the sound source. In the condition with stationary fixation dot, the sound source was designated as moving because phase relative to the coherent visual motion cannot be defined. The sequence of trials from the different conditions was randomized and the direction of the auditory stimulus was counterbalanced for all conditions. The stimuli were presented using “Presentation” Version 9.20 (Neurobehavioral Systems, Inc., Burnaby, BC, Canada).

### Task

Subjects judged whether the auditory sound source was moving initially to the left or to the right. They were instructed to press one of two different buttons to indicate their choice. Responses were recorded with a 5-button fiber-optic response box (Lumitouch, Photon Control, Ltd, Burnaby, BC, Canada). There was no auditory feedback to avoid confounding artifacts with respect to activation in the auditory cortex. Subjects were instructed to wait to the end of the stimulation to respond thereby avoiding confounding artifacts with respect to activation in motor areas. Therefore, response times were measured from the offset of the stimulus to activation of the response button. In all trials, the subjects responded within the 6-s time window allowed. In total, 60 trials were presented, which required a total duration of 15 min.

### Recordings of Eye Movements

During the fMRI measurement, eye movements were recorded to monitor task performance. Eye movements were recorded using the MR-Eyetracker (Cambridge Research Systems, Ltd), a fiber-optic limbus-tracking device [22]. The Matlab Data Acquisition Toolbox was used to acquire the signals derived from the MR-Eyetracker. The sampling frequency of the eye-tracker signal was 500 Hz, the best spatial resolution was 0.1°. The eye-recording system was calibrated with four eccentricities (−10°, −5°, +5°, +10°), to determine the deviation from the fixation position. Using the Matlab Signal Processing Toolbox, we analyzed the eye trajectories offline and evaluated the task performance of the subjects. The evaluation of the eye movements was conducted, due to the relatively small data set, manually with the Matlab Toolbox SPTool.

### MR Imaging

MRI was performed with a 1.5-Tesla clinical scanner (Magnetom Sonata, Siemens, Erlangen, Germany) equipped with an echo-planar imaging (EPI) booster for fast gradient switching and an 8-channel phase array full-head radio frequency receive–transmit headcoil (MR-Devices). High-resolution, sagittal T1-weighted images were acquired with the magnetization prepared, rapid acquisition gradient echo sequence to obtain a 3D anatomical scan of the head and brain. Functional imaging was performed with T2*-weighted gradient EPI. We used a variation of Hall's sparse temporal sampling technique [48], [49] to circumvent interference from acoustic noise created by the gradient coils, such that the onset of the MR acquisition began immediately after the end of the audiovisual stimulation. The acquisition time was 3.3 s, with an adjacent waiting period of 11.7 s, resulting in a total repetition time of 15 s. The time to echo corresponded to 60 ms, the flip angle corresponded to 90°, and we used a field of view (FOV) = 192 mm, with a voxel matrix of 64×64, resulting in a voxel size of 3×3×3 mm. We acquired volumes with 36 slices, aligned parallel to the anterior and posterior commissures (AC-PC) line, with a gap of 0.45 mm between slices and could thus image nearly the entire neocortex, with the only exception of the most anterior part of the inferior temporal cortex. The stimulation protocol for a single experimental run consisted of 60 alternating periods of stimulation and rest, resulting in a total of 60 volumes per subject and 15 minutes scan time).

### FMRI Data Analysis

The data were preprocessed and analyzed on single subject level using Statistical Parametric Mapping, version 2 (SPM2). After motion correction, the functional images were coregistered to the anatomical volume to normalize both to the Montreal Neurological Institute (MNI) Template [50]. Functional images were smoothed with a 3D-Gaussian kernel (full width, half maximum, FWHM = 8 mm). Analysis using the general linear model [51] was done after applying high-pass filtering (cut-off: 128 s). In an epoch design analysis, responses during the 5-s stimulation periods were modeled with a boxcar function convolved with the hemodynamic response function separately for the 3 conditions (fixation moving sound source, SPEM sound source in-phase, SPEM sound source in antiphase). The relevant conditions were contrasted using t-statistics, generating the contrast images for second level evaluation. These images were analyzed on the group level with the SPM2 t-Test for “multiple subjects, one scan per subject”. Voxels surpassing a statistical threshold of *p* = 0.05 (t-contrast analysis, corrected for multiple comparisons, height threshold t = 3) were identified as activated. The SPM2 extension MNI Space Utility (MSU) by S. Pakhomov was used for the identification of anatomical locations. This tool relies on the mni2tal program combined with data of the Talairach daemon [52].
